# Low-cost, mobile EEG hardware for SSVEP applications

**DOI:** 10.1016/j.ohx.2024.e00567

**Published:** 2024-08-10

**Authors:** M. Kancaoğlu, M. Kuntalp

**Affiliations:** Dokuz Eylul University, Graduate School of Natural and Applied Sciences, Department of Biomedical Technologies, Turkey

**Keywords:** Eeg, Ssvep, Bci, Biopotential

## Abstract

The global shortage of integrated circuits due to the COVID-19 pandemic has made it challenging to build biopotential acquisition devices like electroencephalography (EEG) hardware. To address this issue, a new hardware system using common ICs has been designed, which is cost-effective, precise, and easily accessible from global distributors. The hardware system comprises 8-channel inputs EEG hardware with a mobile headset capable of acquiring 5-30Hz EEG signals. First two channels of the design is enabled for steady-state visual evoked potential (SSVEP) operations, and the remaining channels can be powered up as needed. A small 3D-printable enclosure is also designed for the hardware board, which is attached to protective glasses to be used as a head-mounted device. The board includes an additional green LED, 4 pulse width modulation (PWM) outputs for general-purpose input/output (GPIO), 2 buttons for input, and exposed programming pins and digital-to-analog converter (DAC) output from the microcontroller unit (MCU). The proposed hardware system is expected to enable students and young researchers to experiment with EEG signals, especially SSVEP, before investing in professional equipment with the availability of programming codes.

## Graphical abstract

**Figure:**
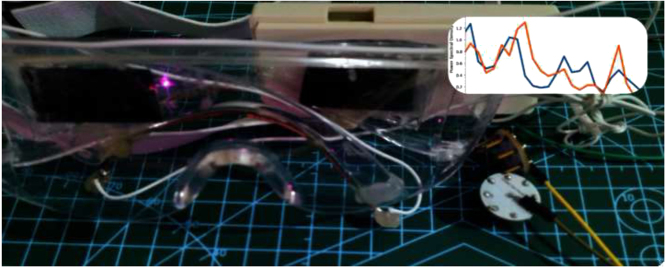

## Specifications table

**Table:** 

**Hardware name**	*EEG Glasses*

**Subject area**	•*Engineering and Material Science* •*Medical (e.g. Pharmaceutical Science)*•*Neuroscience*

**Hardware type**	•*Measuring physical properties and in-lab sensors* •*Electrical engineering and computer science*•*Biomedical engineering*

**Open source license**	*GNU General Public License (GPL) 2.0*

**Cost of hardware**	*100$*

**Source file repository**	https://doi.org/10.17605/OSF.IO/UN5YA

## 1 Hardware in context

In the 1920s, Hans Berger made a groundbreaking achievement by successfully acquiring human brain signals, which led to the discovery of electroencephalography (EEG). This non-invasive, secure, and cost-effective method of brain signal acquisition continues to be widely used in various fields [1]. The temporal resolution of EEG enables easier examination of responses to stimuli. The concept of computer interfacing with brain activity was first introduced by Vidal in 1973 through a publication on brain–computer communication [2]. The advancements in digital recording and real-time signal processing have provided opportunities for the development of brain–computer interfaces for engineering applications, akin to medical diagnosis. Brain–Computer Interfaces (BCI) developed over possibilities of EEG information, leading the possibility of controlling hardware using brain signals. EEG signals carry vital information that can inform a device to start an operation. Visually evoked potentials (VEP) which can be induced over flickering visuals, allows one to send information back to electronics by looking at a visual stimuli for a given time. Well-known phenomena of steady state visually evoked potentials (SSVEP) are one of these methods that works on most of the humans, requires no training and gives high information transfer rate (ITR) with good signal amplitudes. SSVEP works by gazing at flickering frequencies above 3 Hz, with the frequency and phase information generates time-dependent responses on occipital lobe [3]. As an alternative, low frequency blink stimulations causes a form of signal which has a peak at around 300 ms after the signal observed. This peak signal named P300 is captured and a part of another method called P300 BCI systems [4]. Hybrid systems exists as well, using both methods for increased performance [2].

Brain–computer interface (BCI) systems using EEG signals have a lower success rate compared to smart prosthesis systems utilizing electromyography (EMG) signals [5]. This is primarily because the amplitude of EEG signals is weaker and they are more susceptible to noise, which significantly impairs their performance. Under certain conditions, it could be challenging to utilize muscle-based communication with patients. A notable example is locked-in syndrome(LIS), where patients may only have the ability to communicate through eye movements, if even possible [6]. BCI becomes best option for such cases [7].

For our study, we have chosen the steady-state visual evoked potential (SSVEP) as our preferred method. This choice is due to its high information transfer rate (ITR), greater amplitude, and the absence of the need for training [8]. The SSVEP signal predominantly originates from the occipital lobe, which is located at the posterior part of the head. Although it is possible to acquire SSVEP from the ear channel, the signal amplitudes significantly decrease in this case [9], [10].

In our study, we opted to utilize an LED-based blinking system as opposed to a computer screen due to considerations such as latency and the ability to achieve different frequencies [11]. However, it is important to note that there will be no direct connection between the EEG device and the stimulus hardware, resulting in the loss of phase information. If necessary, the EEG hardware can employ a Bluetooth connection to address this issue. While many researchers use computer monitors for stimulation, there are also open-source alternatives utilizing LED technology for SSVEP stimulation [12].

For a BCI device to function effectively, it requires a suitable headset. Clinical research-grade headsets with wet electrodes are commonly used in research settings. However, in everyday usage, they necessitate tedious preparations to be practical. In this case, we have utilized dry electrodes, which have shown comparable performance depending on the substrate material used [13]. Non-contact capacitive active electrodes with built-in circuitry offer greater flexibility but have higher impedance. Additionally, carbon nanotubes have been employed as electrodes in certain cases [14]. Nevertheless, traditional AgCl electrodes remain widely used due to their performance [15], [16]. OpenBCI’s hardware with “Ultracortex Mark” headset, featuring dry electrodes, provides an open-source alternative, although it covers the entire head, which may limit portability. In-ear or around-ear electrodes have shown promise for movement and social research, offering advantages in terms of mobility and social acceptability [17].

With advancements in analog hardware quality, there is been a surge in the development of custom EEG systems by both researchers and hobbyists, extending to EEG headsets. The widely utilized ADS1299 serves as a compact hardware solution [18]. Typically, developers integrate bandpass and notch filters to reduce AC noise, enhancing signal quality for subsequent amplification stages. The AD620 is renowned for its effectiveness in biopotential acquisition, frequently featured in open-source designs. Alternatively, the INA128 instrumentation amplifier offers comparable performance [19], [20], similarly boasting high voltage input ranges exceeding 18 V, facilitating higher pre-gain stages for superior signal fidelity.

In common practice, EEG signals undergo extensive filtering and amplification of the AC component, preparatory to conversion by 12–16 bit ADC hardware. Designers often have preferences regarding the device’s configuration, with some opting for a stationary box setup during acquisition, while others, like ourselves, integrate it into headgear [21], [22].

Our hardware is designed to be a portable device powered by a battery, utilizing a 3.3 V power supply for both digital and analog interfaces. The use of low voltage imposes certain limitations on the available hardware options, as components need to meet minimum voltage requirements. Meanwhile, low voltage hardware offers the advantage of requiring fewer components and enabling smaller footprints. Conversely, 5 V hardware powered by a 4.2 V LiPo battery would necessitate the integration of charge-pump or inductive boost circuits, complicating the power section. Additionally, inductive power hardware may cause voltage spikes during startup, which can lead to challenges during the verification process for commercial production. This hardware, powered by a single LiPo or a coin cell, and even a power bank if necessary, employs just one linear regulator for energy management, ensuring versatility and portability. Careful selection of low-noise hardware is crucial, as each active or passive component can introduce additional noise. In order to optimize the PCB size for mobility, we have designed a 4-layer PCB, utilizing both sides for component placement. For the sake of simplicity, we have not implemented impedance or gyroscope functionality. However, the board can be slightly enlarged to accommodate additional sensors if desired, although they are not necessary for SSVEP.

In the commercial market, there are various EEG hardware options available, such as the single-channel Neurosky, the multi-channel EMOTIV with a monthly subscription [12], the open-source OpenBCI, and the multi-channel Muse, which is geared towards meditation practices. Mentalab Explore, as another option for consumer hardware also available for clinical EEG acquisition [23]. These commercially available options are priced under 1000$, and while our hardware is competitive in terms of signal acquisition, it serves as a more affordable entry point for researchers and engineers to embark on their EEG projects before investing in or developing more expensive hardware. It is important to note that the frequency band of our hardware is lower than that of verified hardware, and it does not comply with IFCN (International Federation of Clinical Neurophysiology) standards as described in Table 1 [24], [25].

**Table 1:** Common EEG standards and our hardware [25].

	Input range	Input referred noise	Bandwidth	Electrode offset	Input impedance @50/60	CMRR @50/60	Safe current
IEC60601	$0.5mV_{pp}$	$6\mu V_{pp}$	0.5–50	300mV			50μA
IFCN		$0.5\mu V_{rms}$	0.16–70		>100MΩ	110 dB	
Our Device	$210mV_{pp}$	$10\mu V_{pp}$	2–45^a^		100GΩ	95dB	40μA

a Exact low-pass value depends on filter. We use nyquist, however, it is obvious that device cannot acquire high frequencies with DIY electrodes due high amount of noise.

## 2 Hardware description

Analog front ends (AFEs) play a crucial role in biopotential applications as they offer substantial benefits by reducing the need for additional hardware and providing satisfactory signal quality for development purposes. While custom-designed solutions are available, utilizing a single-chip AFE offers convenience at a slightly higher cost. However, it is worth noting that due to the popularity and demand for such AFEs, they may frequently go out of stock, resulting in developers having to wait for restocking before they can proceed with their projects. The ADS1299 is a widely used AFE that meets the standards for EEG acquisition [26]. We went for a custom design utilizing instrumentation amplifiers and analog to digital converters (ADC) commonly available to overcome shortage and design in lower cost. A similar hardware is used in OpenBCI ganglion board with design files are available in github .

In our focus on engineering applications related to SSVEP, we prioritize keeping the frequency range of bandpass filters as narrow as possible. Throughout our development process, we have found that only DC filtering is necessary, and passive lowpass filters can be either avoided entirely or selected based on the Nyquist frequency. Due to the high impedance of our electrodes, our hardware tends to pick up a significant amount of AC noise, making the use of a lowpass filter with a lower frequency highly advantageous.

While the hardware DC filter aids in compensating for the high offset of the instrumentation amplifier, there still remains a DC offset of approximately 3 mV associated with the inexpensive LM324LV. Furthermore, the half-cell potential observed with DIY electrodes can lead to the DC filter not functioning optimally, resulting in a slight offset.

To mitigate these challenges, we employ minimal amplification and utilize high-quality ADCs to ensure the acquisition of EEG signals.

**Fig. 1:**
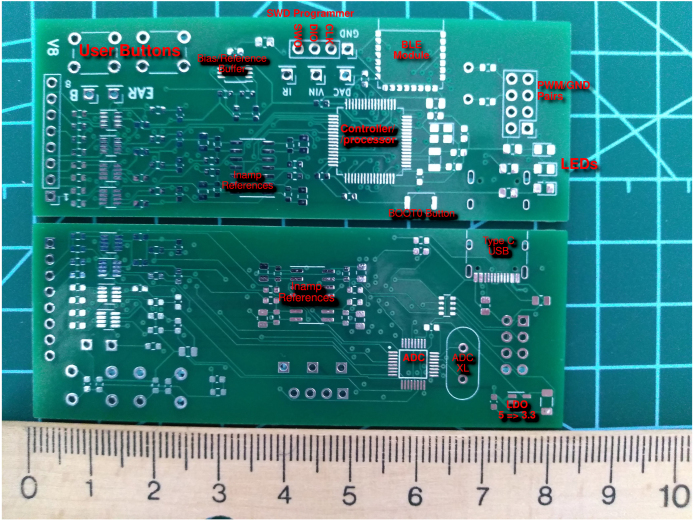
Unsoldered card with descriptions.

Hardware in biomedical signal acquisition is getting smaller and more mobile, into watches and cellphones. In our setup we went for a smaller hardware that can be carried around using external battery and BLE system. Our design is shown in Fig. 1.

**Fig. 2:**
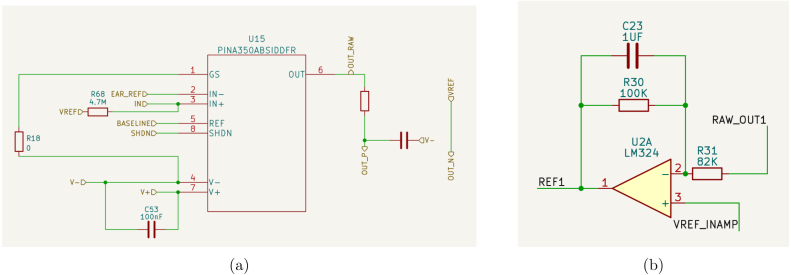
Analog circuit: (a) Instrumentation and filter before ADC (b) Inamp reference.

**Fig. 3:**
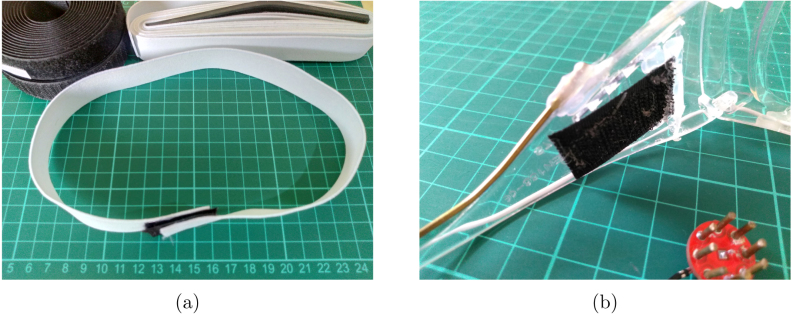
Headset hardware: (a) Headband for electrodes (b) Brass glued on glasses.

• **Electrodes** In EEG acquisition systems, the electrodes play a crucial role as they are responsible for capturing the EEG signals. During the early stages of EEG technology, the signals were recorded on paper, highlighting the significance of electrodes in the process. AgCl electrodes currently dominate the commercial market as they are considered superior to many other substrate materials [27]. It is important to note that even with a low-precision system, such as 10-bit ADC Arduino Uno, the EEG acquisition will work fine while a 24-bit high-precision system fail if unsuitable electrodes are used. In our specific setup, we have acquired small electrodes for channel acquisition, while we have constructed brass electrodes for ground/reference purposes. Although the use of brass electrodes introduces additional noise, it provides a cost-effective and straightforward solution by attaching a brass rod to protective glasses. You can refer to Fig. 3(b) to visualize our electrode setup on the protective glasses.
• **Amplifiers** In order to maintain simplicity in our design, we have exclusively utilized instrumentation amplifiers (inamps). Due to the presence of 1/f noise in EEG signals, careful consideration must be given to the selection of inamps to avoid introducing additional noise into the circuit, as they contribute to the overall input level noise. The options for inamps are limited to low voltage chips, and those with low 1/f noise characteristics are preferred. EEG measurements typically require high impedance for dry electrodes, which requires a balance between bias current and 1/f noise. While it is possible to construct an instrumentation amplifier using operational amplifiers (opamps), such a design would result in significantly lower common-mode rejection ratio (CMRR), making it more susceptible to AC noise. However, the INA350 inamp stands out in its price range, as shown in Table 2, and offers the advantage of not requiring any additional components. Despite having slightly higher noise characteristics, the INA350 is a preferred choice for building an affordable EEG device. It is important to note that the high AC noise and electrode potentials in our application impose a limitation on the maximum gain. INA350CDS is used with 30 gain in this device. The LMP7732, a dual operational amplifier (op-amp), plays a crucial role in buffering the reference voltage from the analog-to-digital converter (ADC) and facilitating the primary bias return circuit. Alternatively, any 8-pin VSSOP package with standard dual op-amp pin configurations (negative on the 4th pin, positive supply on the 8th pin) can be replaced with this integrated circuit (IC). It is recommended to opt for a device exhibiting very low offset voltage and minimal 1/f noise for optimal performance.
• **Analog to Digital Coverters (ADC)** The EEG signal is predominantly an alternating current (AC) signal, and electrodes introduce significant offset. Table 3 provides an overview of various options available with the ADS1299 Analog Front End (AFE), which offers suitable solutions for amplifying and capturing these signals. Similarly, the CS5534 also features instrumentation amplifier inputs, eliminating the need for additional inamp components, thereby saving both space and costs. For our device, we have utilized the ADS131 series, which is an affordable and precise amplifier series. The ADS131M models, specifically, operate with a 3.3 V analog input and are fully differential, ensuring high precision in signal acquisition. Analog circuit used per channel for this ADC is shown in Fig. 2. The negative inputs of the inamp are connected to the ear reference. Both ear and electrode inputs are linked to the reference for bias current using megaohm resistors. Optionally, a zero ohm resistor can be used, soldered to achieve a gain of 30. In this setup, each negative input into the ADC is guided by the reference, while the positive inputs are filtered to prevent aliasing. Additionally, the reference of the inamps is driven through highpass filters, effectively reducing offset to a significant extent. This ADC handles out of band frequencies during sampling, thus RC lowpass/antialiasing filter can be selected as high as 8 kHz. Moreover, we use an antialiasing filter to filter out high-frequency signals, compensating for the fact that the instrumentation amplifier cannot handle frequencies above 40 kHz. It is up to the designer to use pick values for filtering, in our setup, we went for R=2 K C=10NF for following datasheet frequency (8 kHz). We recommended to use R=6.2 K and C=220NF for nyquist frequency or lower in Fig. 2(a). This ADC uses SPI protocol to communicate with microcontroller. Before starting conversions, settings are written into ADC registers for EEG operation, including sampling rate, used channels, power mode and so on.
• **Microcontroller** A brain–computer interface (BCI) system is tasked with processing EEG data and performing an associated action. To address security and portability concerns, the EEG device itself processes the results of an SSVEP-based system. This approach requires substantial computational power, thereby limiting the range of available algorithms. The Welch method is employed for signal processing, and the obtained results are transmitted using Bluetooth Low Energy (BLE) or USB. To facilitate the processing tasks, a microcontroller unit (MCU) with Floating Point Unit (FPU) support is utilized to handle floating-point computations required during intensive Fast Fourier Transform (FFT) calculations. The processing capabilities are augmented through the use of the CMSIS-DSP library. In our particular system, the STM32L433 microcontroller is chosen due to its high performance and low power consumption. The 64-pin package provides multiple interfaces for various functionalities, although it is possible to opt for the reduced 48-pin package if necessary. While the STM32F103 is widely used and available, particularly with the blue-pill board, it is advisable **not to use F1 and L1 series** due to their inability to perform hardware floating point operations. It is worth noting that STM32 chips with M3-Cortex cores lack FPU unless explicitly stated. For tasks involving dense math operations, opting for STM32 M4-Cortex or M33-Cortex series chips is recommended, as they are specifically designed to handle such operations effectively [28], [29]. While our setup enables real-time computations, there is an option to disable the BCI mode. This allows for the collection of raw EEG signals for offline analysis. In cases where you need to work with EEG data instead of flickering frequencies, our software offers a solution to obtain raw data by adjusting the firmware settings.
• **Bluetooth Low Energy (BLE)** Hardware that uses radio signals requires thorough verification before it can be used. This verification process can be more expensive compared to entire hardware. To make this process smoother, there are modules available with FCC/CE certifications that meet the required standards. For communication purposes, a Bluetooth module with FCC/CE verification is sufficient. In our case, we chose the E104-BT05 module, which is affordable and comes with pre-installed firmware and an AT command set. We bought this module from a Chinese vendor, but you can use a different module if needed. The module’s small size allows for efficient use of space on the PCB.
• **Brass Electrodes** For the purpose of reference/ground electrodes, a 1.5 mm brass rod is employed. Instead of utilizing earclips, these electrodes are positioned directly on the ear, which may introduce movement-induced noise. The attachment of wires to brass electrodes is facilitated by tightly wrapping them and subsequently soldering. The electrodes are bent and fixed to glasses using hot glue. Brass, being an average material for biopotential acquisition, offers a cost-effective solution. It is important to acknowledge that certain brass alloys may contain lead, prompting owners of 3D printers to avoid common brass nozzles for food-safe printing. However, in our particular scenario, the brass electrodes will be used on the skin. In addition to their application as flat electrodes, brass electrodes are also employed for cap electrodes. While alternative professional electrodes offer superior options at a low cost, our development specifically utilizes brass electrodes. It is important to note that brass electrodes exhibit a significant offset, which necessitates the implementation of high-pass filters by designers. Brass is softer than steel, making the cutting process of brass electrodes much easier. Special tools can be used to achieve the desired sizes for brass electrodes. However, cap electrodes require additional processing. To flatten the tips, a dremel tool is used. Any tool that can effectively work with soft metals can be used for this purpose. While it is not mandatory to use a custom coating, it is recommended to have a round tip configuration for the electrodes. This helps to improve contact and reduces the risk of skin lacerations during electrode application. The ear electrodes have a length of approximately 10 cm, while the cap electrodes are cut to around 5 mm. The developers have the flexibility to adjust the length of the electrodes as they see fit to enhance the signal quality according to their requirements.
• **Headsets** Hardware manufacturers often develop proprietary headsets specifically targeting their own hardware. These headsets are designed with fixed electrode positions, simplifying the processing of signals from each electrode. Customized hardware can take advantage of these fixed positions to enhance functionality. For instance, electrodes placed on the forehead can be used for processing signals related to relaxation. Fixed occipital lobe placements are employed for SSVEP/P3 stimulations. Alternatively, some hardware designs utilize band-like headsets that allow for electrode placement at various locations, enabling users to leverage the manufacturer’s API for evaluation purposes. In-ear electrodes also have fixed positions; however, acquiring reliable signals from ear electrodes has certain limitations, as previously discussed. Our design involves a straightforward approach using a band-based headset that is worn over protective glasses to acquire occipital signals. For SSVEP operations, a single electrode attached to a velcro band proves sufficient. The velcro band is hot-glued to a elastic fabric, which serves as a means of securing the electrode. Initially, we attempted to attach the velcro band directly to the glasses; however, we found that it provides a more secure hold when fastened around the head.

**Table 2:** Instrumentation amplifiers possible for EEG.

Name	0.1–10 Hz Noise G=10 (μV)	Price of 1 (100 pcs)	Impedance	Input offset voltage
AD620	0.8	8	10G	125u
AD8237	1.5	2.2	100M	75u
AD8553	0.7	3	10G	50u
MAX4208	2.5	3.2	2G	20u
INA350	3.2	<0.5	100G	1.2 m

**Table 3:** Some ADC/AFE options for EEG acquisition.

ADC	Manufacturer	Gain Max	Frequency	Noise (μVrms)	Supply voltage	Non-Mux inputs	Differential	ESD	Notes
AD7779	Analog Devices	8	1000	1.33	3.3	Y	Differential Pseudo Differential Single Ended	+	EEG, Daisy-Chain

ADS1256	Texas Instruments	64	2000/8	4.025	5	N	Single Ended Differential (N/2)	+	Buffer

CS5534	Cirrus Logic	64	960/4	8.34	3.3,5	N	Differential	–	Programmable Instrumentation Amplifier

ADS1299–8	Texas Instruments	24	250	1.08	5	Y	Differential Pseudo Differential Single Ended	–	Lead Off Detection, Bias Output, Analog Frontend, Daisy-Chain

ADS131M08	Texas Instruments	128	250	1.90	3.3	Y	Differential	+	Current-Detect Mode

## 3 Design files

All design aspects of our project were accomplished using freely available software. The enclosure design was created using FreeCAD (www.freecad.org), a powerful open-source 3D CAD modeling software [30]. For the PCB design, we utilized KiCAD (www.kicad.org), an open-source electronic design automation (EDA) suite. We have uploaded the project files, allowing for further adjustments and fine-tuning as needed.

### 3.1 Design files summary

The provided files are in the format of KiCAD projects, and the corresponding holder plastics have been designed using FreeCAD. It is worth noting that the entire software development process has been carried out using free tools under GNU/Linux. In cases where LED stimulation hardware is already available, there is no need to build an additional LED interface.

**Table 4:** Design files.

Design filename	File type	Open source license	Location of the file
KiCAD for Board	Design Diagram	GPLV3	10.17605/OSF.IO/UN5YAgithub latest firmware

KiCAD for LED	zipped KiCAD Project	GPLV3	10.17605/OSF.IO/UN5YA

KiCAD for Electrodes	zipped KiCAD Project	GPLV3	10.17605/OSF.IO/UN5YA

FreeCAD design for EEG & LED Box	zipped FreeCAD design	MIT	10.17605/OSF.IO/UN5YA

The electrode design incorporates small round PCBs with 2.5 mm through-hole (THT) openings that facilitate the connection of brass rods. The central hole of the PCB is utilized for soldering a Dupont cable, enabling the electrical connection for signal transmission.

Design files are uploaded in separate archives as shown in Table 4.

## 4 Bill of materials

• **LED flicker** Our SSVEP stimulation solution utilizes a MOSFET circuit to drive flickering LEDs. By employing a PWM generator such as the NE555 or a microcontroller, the MOSFET circuit is capable of controlling the flickering pattern of the LEDs. To ensure proper operation, we have incorporated 1 W LEDs, 1 W capable through-hole resistors (THT), and two separate resistors for grounding the MOSFET’s gate. This configuration operates on a 5 V power supply. Fig. 6 shows our setup with two LEDs and Table 5 shows design files for 4-LED flicker table.
• **Electrodes** While it is generally recommended to purchase dry electrodes from reputable medical vendors for optimal quality and reliability, it is possible to construct your own electrodes using brass rods for experimental purposes. However, it is important to note that brass can oxidize over time when exposed to moisture, which can affect the electrode’s performance. Therefore, it is crucial to keep the homemade electrodes dry and properly stored to minimize oxidation and maintain their functionality. PCB-based brass DIY electrode files are described in Table 6.
• **Head Band** To create a comfortable headband for electrode placement, we utilized elastic fabric and a Velcro band shown in Fig. 3(a). While it is possible to glue the Velcro band inside protective glasses, as depicted in Fig. 3(b), we opted to design a round headband specifically for occipital signal acquisition. This headband design ensures a secure and adjustable fit, allowing for reliable electrode placement on the occipital region.
• **Headset** To facilitate the development of the headgear, we utilized a cost-effective protective headset as a base tool. Any glasses that have the possibility of inserting a brass rod can be used in conjunction with this hardware. The Velcro band is securely glued to both the EEG box and the glasses, ensuring a stable and adjustable fit. By providing an external power supply, the headset becomes completely mobile, allowing for greater flexibility during use. However, it is important to note that without an accelerometer, the headset lacks movement information tracking capabilities. Necessary headset parts are shown in Table 7.
• **PCB** The centerpiece of our headset is the compact 8-channel EEG board, which plays a vital role in signal acquisition. Despite its small size, this board is densely populated with components on both sides, allowing for optimal utilization of space. To enable wireless functionality, we have integrated a small Bluetooth Low Energy (BLE) module into the design, facilitating seamless communication between the headset and other devices. The microcontroller unit (MCU) with a hardware floating-point unit (FPU) plays a crucial role in executing the necessary code for EEG analysis. To optimize the processing capabilities of the specific microcontroller architecture, we leverage the CMSIS-DSP library, which offers a compact set of functions designed for efficient execution on the MCU. This MCU-based processing approach, although limiting in terms of signal processing and classification options, provides a cost-effective solution for our EEG system. To ensure accurate signal acquisition, we utilize the ADS131MX series, which are 3.3 V analog-to-digital converter (ADC) units with 24-bit precision. While the price-performance ratio of these ADC units is favorable, it is important to note that the M-series models only support 3.3 V analog inputs. In our battery-powered system, which operates on a single low-dropout 3.3 V regulator, this aligns perfectly with our voltage requirements. If modifications are made to the board layout, careful consideration should be given to the placement of decoupling capacitors to maintain optimal performance. In terms of the PCB design, we have incorporated a small box that serves the purpose of securely mounting the PCB onto the glasses. This feature enhances the convenience and practicality of the overall headset design. The Welch algorithm is employed in our system to analyze the frequencies associated with steady-state visual evoked potentials (SSVEP). Specifically, we focus on evaluating the alpha band and the frequencies corresponding to the flickering stimuli. The board consists of 8 × 1 channel inputs, bias, and ear reference pins, as well as SWD programming pins, voltage, infra-red output, and DAC output pins for operation. Additionally, there are three LEDs: green, blue, and red, used to indicate the device’s ongoing state. The red LED indicates an active channel that is saturated, possibly due to disconnection or high offset. The blue LED indicates a Bluetooth connection, and the green LED remains always on to show that the device is running. Just below these LEDs, there are 4 × 2 pin pairs for PWM and ground, intended for operating SSVEP based LED stimulus. However, this feature is not used and can be repurposed for additional communication needs. It is important to note that the PWM output heavily affects the signals as they pass near the channel cables.

**Table 5:** LED stimulation components.

Component	Number	Cost per unit currency	Total cost	Source of materials	Material type
1W LED^a^	4	1$	4$	Aliexpress (see below)	LED

THT Resistor	12	<1$	2$	Local reseller	Passive

2N7002K SOT-23	4	1$	4$	Local reseller	MOSFET

PCB	10	2$	20$	jlcpcb.com	PCB

Any NUCLEO board (optional for PWM)	1	15$	15$	ST eStore	Development Board

Black filament	30 meters	1$	5$	Any filament source	Polymer

Cable^b^	40 meters 0.22 mm${}^{2}$	1$	5$	Any source	Wire

5V 2A Adapter^c^	1	5$	5$	Amazon	Electronics

a The specific source of the LED is ( https://tr.aliexpress.com/item/33026779778.html). However, it is worth noting that these LEDs are widely available and can be obtained from various sellers since they are commonly used. For optimal amplitude, we recommend using white-colored LEDs in the SSVEP stimulation setup.

b Please consider purchasing a multi-wire cable that is thicker and capable of carrying a current of over 100 mA. Dupont cables may not be suitable for this requirement due to their limited current-carrying capacity.

c Any suitable adapter will work for this setup, including a metal chassis adapter or a USB charger. Ensure that the chosen adapter meets the necessary specifications, such as voltage and current requirements, to safely and effectively power your device.

**Table 6:** DIY electrode parts.

Component	Number	Cost per unit currency	Total cost	Source of materials	Material type
1.5 mm brass rod (50 cm)^a^	1	5-10$	10$	Local reseller*	Metal rod

PCB^b^	5	2$	20$	JLCPCB	PCB

AgCl Dry Electrodes^c^ (optional)	30	50$	50$	OpenBCI shop	Electronics

a The availability and pricing of brass rods can vary depending on your local area. Factors such as location, supplier availability, and market demand can influence the accessibility and cost of brass rods. It is advisable to explore local hardware stores, metal suppliers, or online marketplaces.

b For PCB manufacturing, we utilized JLCPCB, which provides cost-effective solutions for producing PCBs. Consolidating the order and purchasing all the PCBs at once can help reduce shipping costs. The mentioned price is specifically for 2-sided PCBs used as holders for the brass electrodes, with diameters of 20 mm and 15 mm. By ordering multiple PCBs together, you can take advantage of potential cost savings in shipping expenses.

c OpenBCI offers affordable dry electrodes that are marketed as having a lifespan of approximately 10 uses. These electrodes are competitively priced compared to other hardware options, making them a cost-effective choice for performance purposes.

**Table 7:** Headset parts.

Component	Number	Cost per unit currency	Total cost	Material type
Protective Glasses	1	5$	5$	Plastics
Velcro band	5mt	5$	5$	Cloth
Elastic fabric	10mt	5$	5$	Cloth

**Table 8:** PCB components.

Component	Number	Cost per unit currency	Total cost	Material type
SMD Capacitor^a^ (15PF, 30PF, 100NF, 220NF, 1UF, 47UF)	100	1$	10$	SMD

SMD Resistor^b^ (3.9 K, 5.1 K, 10 K, 18 K, 22 K, 82 K, 240 K)	100	1$	10$	SMD

EByte E104-BT05	1	5$	5$	SMD Module

STM32L433RCT^c^	1	5$	5$	MCU

INA350	8	0.5$	5$	inamp

LM324LV	2	0.5$	1$	opamp

TLV73333PDBV	1	0.5$	0.5$	regulator

LMP7732^d^	1	2.5$	2.5$	2-ch opamp

ADS131M08	1	8$	8$	ADC

4-layer PCB^e^	5	10$	10$	PCB

a Have a set of SMD 0805 package capacitors. Filters can be tolerated. Check bom html file generated by KiCAD for details.

b Multiple sets of SMD resistors are cheaper than capacitors. It is advised to buy a set of 0603 resistors with % 1 tolerance.

c Requires SWD programmer. We used ST-LinkV3.

d Check Amplifiers section for alternatives.

e Includes all the remaining parts like connectors, crystals and buttons.

## 5 Build instructions

**Fig. 4:**
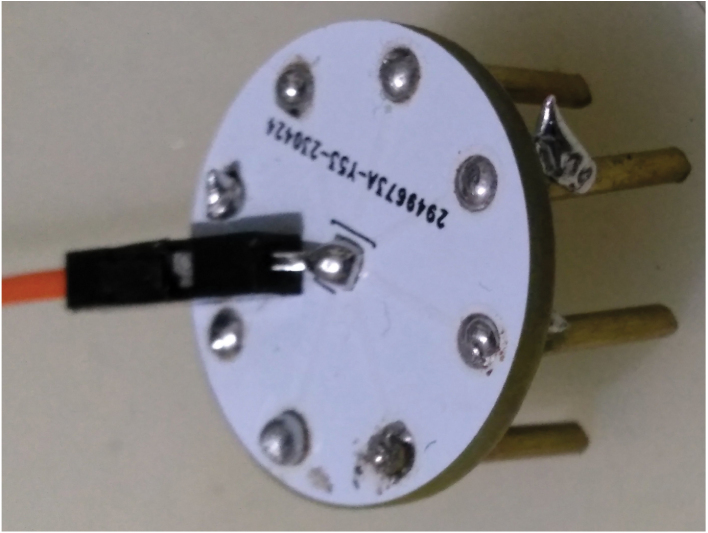
2 cm diameter PCB electrodes with lead-free solder.

**Fig. 5:**
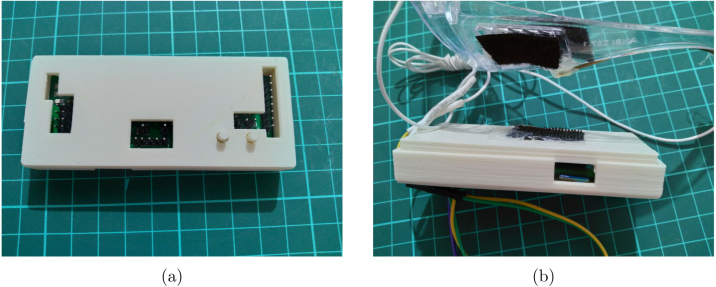
PCB box assembled (a) and mounted (b)

**Fig. 6:**
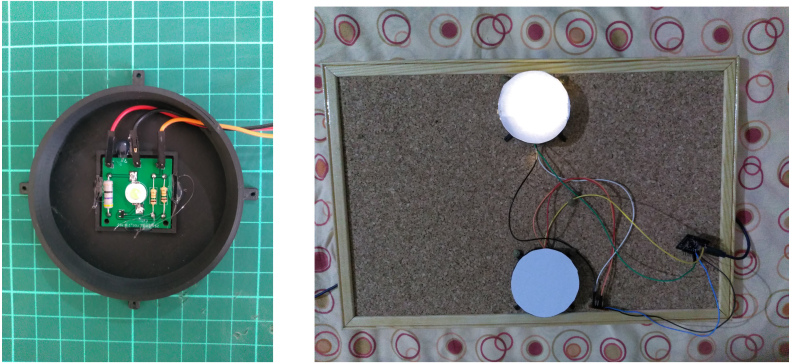
SSVEP testing hardware, 3D printed enclosure for 1 W LED and setup.

**Fig. 7:**
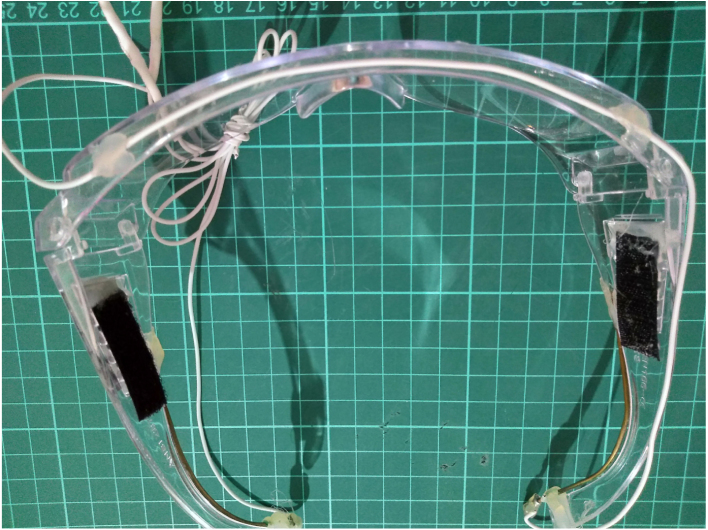
Brass rods and wires on glasses.

• **Electrodes** The PCB electrodes utilize brass rods, which cannot be easily soldered using regular tin solder. However, an alternative approach involves filling the PCB holes with tin solder, inserting the brass rods carefully, and completely filling the backside of the PCB to create a solid enclosure that securely holds the electrodes in place. The alignment of the brass rods does not need to be perfect, allowing for some tolerance in the assembly process. Fig. 4 showcases a fully assembled electrode featuring 1 cm long and 1.5 mm thick brass rods. Take note of the fillings on the backside, as they contribute to the electrode’s stability. It is important to ensure that the backplate is covered adequately to prevent any looseness. Finally, a dupont cable can be connected to the middle through-hole for establishing the electrical connection. Using thicker brass rods for the PCB electrodes may necessitate larger holes in the PCB. However, it is worth noting that thinner 1 mm rods may be prone to bending during usage, as brass is a relatively soft metal. Additionally, thicker rods would require more pressure to make contact with the skull, particularly when there is hair in between. This consideration should be taken into account when selecting the appropriate thickness for the brass rods used in the electrodes.
• **EEG Board** When soldering small components such as 0805 or 0603, it is recommended to have prior experience with surface-mount soldering techniques. Using a hot air gun can facilitate the soldering process, especially for soldering SMD modules. While using a stencil for solder paste application would be beneficial, in this prototyping stage, we opted for soldering the components manually on a single board for testing purposes. Parts are shown in Table 8. You can solder as many channels as needed for your specific requirements. Leaving some inamps unsoldered will not have any adverse effects on the circuitry. If you have access to a 3D printer, you can utilize the provided files to print out the box and two buttons. Once printed, the box can be joined with the glasses by attaching them together using a glued velcro band as shown in Fig. 5(b). This assembly will help secure and integrate the headset components for convenient usage.
• **Flickering LEDs** As an optional component, our flickering LED setup utilizes 1 W LEDs and requires a 3D-printed enclosure. To assemble the setup, first print plastic part and assemble PCB. Circle has a diameter of 7 cm, while the LED PCB has dimensions of 27 × 27mm. You can place the PCB inside the stand and secure it with glue. Finally, cover the front of the setup with a semi-transparent material like paper to diffuse the light. The assembled LED setup is illustrated in Fig. 6. As mentioned earlier, these components are not necessary for the EEG device itself, but they are required for SSVEP stimulations used during verification. Please ensure that you properly connect and join the final cables and connect them to an appropriate adapter. Power cables should stay connected to 5 V power and it should share ground pin with controller circuit. We developed a small STM32F070 based board for USB connection and powered system using computer USB port. Multiple 2.54 mm pin pairs are joined together and each LED is powered by these power pins.
• **Headset** We designed our headset with a protective glass to house the EEG hardware and ear connections. As brass does not readily stick to regular solder, we tightly wrap the wires around the brass rods and solder them in place. After soldering, we bend the wires to fit the curves of the ears on the glasses. For proper functioning, we use longer cable for the bias current connection. To complete the assembly, we glue the cable on top of the glasses. The final result can be seen in Figure Fig. 7.

## 6 Operation instructions

After completing the PCB and plastic assemblies, the user needs to decide which channels to use and upload the firmware using programmer tools. The active channels are hardcoded into the firmware, but with an updated firmware, the user can have more flexibility and control over the settings using extra buttons on the PCB.

To set up the device:

**Fig. 8:**
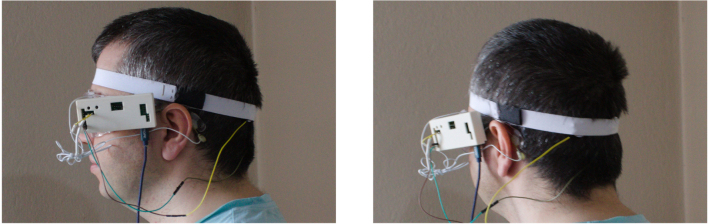
Wearing headset.

• If you have a custom design headset with electrodes, you can bypass glass/band setup and connect electrodes to dupont cables directly. Commercial electrodes will yield better performance than brass setup.
• Wrap the elastic band around your head and place the electrodes under it. The lightweight electrodes will stay in place even during movement. The recommended positions are in the occipital region, but you have the freedom to choose the lobe for acquiring signals. For SSVEP, it is suggested to place the electrodes on O1, O2, or Oz positions.
• Wear the protective glass headset and ensure that its brass rods touch your ears. Make sure that there are no obstacles, like hair, blocking the connection.
• Connect the device to a computer or USB power source. It will be recognized as a serial port on the computer, so any software capable of acquiring data from serial ports can easily retrieve EEG data in text format. If a power source is used, connect to onboard BLE server using a client, preferably an ESP32 development board programmed for this purpose.
• Check that the red LED is not active. The device checks for saturation on active channels twice every second and updates the LED status accordingly. An active red LED indicates that at least one channel is saturated. Ensure proper skin contact and clean the skin if needed. Although dry electrodes have high impedance, good skin contact is necessary to acquire EEG signals.
• To see EEG channel data, you can use Arduino IDE serial tools. Each line contains active channel data in text format with spaces for separation, making it similar to comma-separated values (CSV) files. With just a few lines of Python code, user can acquire serial data and save it to a file for offline EEG operation.

Setup is shown in Fig. 8. You have the option to activate BCI mode, which acquires data for a specified length of time to inspect frequency dominance in hardcoded frequencies. However, this will temporarily disable EEG output since ADC acquisition stops during processing. For offline operation and recording data, it is recommended to keep BCI mode off and save the data to a file.

## 7 Validation and characterization

For discovering energy consumption, device connected to PC using FNIRSI-FNB58 power meter.

We observed a current peak of 60 mA during 8-channel operation, with the MCU estimated to consume up to 60 mA during computational tasks. Given its low-power requirements, this device can be effectively powered by small LiPo batteries. During communication, the minimal power consumption is measured at 33 mA. Comparatively, BLE transmission demands less power than USB, though it is noteworthy that the BLE module consumes energy even in the absence of active operations. Our tests reveal that USB operation without the BLE module soldered consumes 28 mA, indicating that the BLE module draws approximately 7 mA of current onboard. While it is feasible to put the BLE module into sleep mode to conserve some power, the reduction of AC noise through an isolated power supply proves to be more advantageous.

**Table 9:** Current consumption(mA) for 2-Ch EEG acquisition.

		**USB ON**	**USB OFF**
	**BLE ON**	43	33
	**BLE OFF**	35	30

We conducted detailed current measurements during the 2-channel test stage, as outlined in Table 9.

**Fig. 9:**
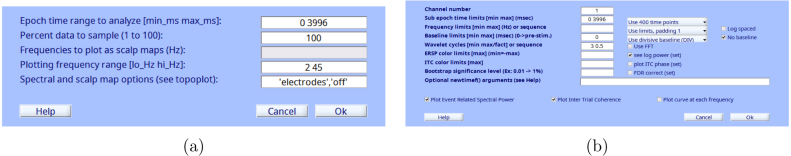
EEGLAB Settings for: (a) Spectra and maps (b) Channel-time frequency spectrum.

Validation of EEG signals was carried out using EEGLAB version v2020.0 compiled for GNU/Linux. The tests and data acquisitions were performed on an Arch-Linux distribution. The settings utilized for generating graphs are depicted in Fig. 9. Both spectrum and frequency analyses were conducted and compared against their respective alternatives. It is important to note that no signal preprocessing was applied to the records.

Three volunteers, aged between 35 and 55, participated in these validation trials. Each trial captured around 10 s of records, and we selected 4 s of samples from these recordings. The sampling rate was set at 250 Hz, and every record comprised 1000 samples. We used the Welch method for analysis, with an overlap of 10 samples. Volunteers were gazing at the flickering lights around 30–50 cm distance.

While clinical EEG recordings typically require low impedance levels below 10 Kohms, our setup deviated significantly, with brass electrodes registering impedance levels ranging from 1–2Megaohms. This renders our setup unsuitable for clinical applications. However, despite this limitation, our setup effectively captured SSVEP responses, enabling real-time BCI operations.

We gathered signals from the O1 and O2 positions. We calculated the average in the time domain and employed this average for subsequent calculations. This led to frequencies extending across a range of frequencies. Each test contains 1000 samples in 250 Hz, a total of 4 s.

One common method of studying EEG signals is by observing the alpha wave. These waves tend to increase notably when an individual is relaxed or has their eyes closed. However, when higher noise levels are present, particularly from AC sources, visually confirming the signal becomes difficult. Hence, we use frequency distribution of EEGLAB, as discussed earlier. Alpha waves are most prominent in the 9–12 Hz frequency range, providing us valuable insights into the obtained signal. Despite its higher impedance, our hardware displays a lower overall signal-to-noise ratio (SNR).

**Fig. 10:**
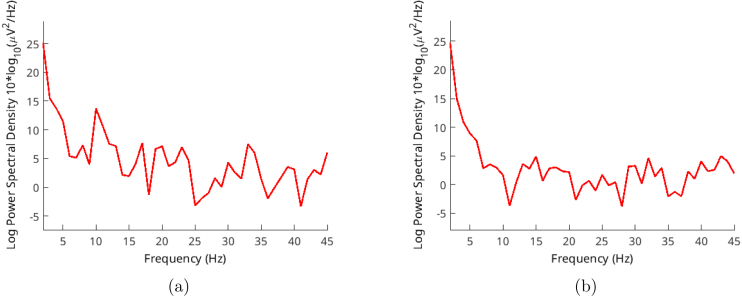
Volunteer-1 alpha test for (a) closed (b) open eyes.

**Fig. 11:**
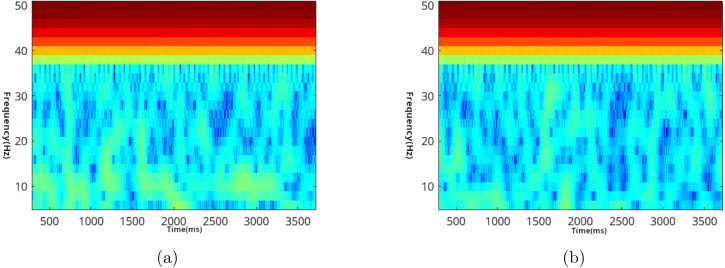
Volunteer-1 alpha test spectrum for eyes (a) closed (b) open.

**Fig. 12:**
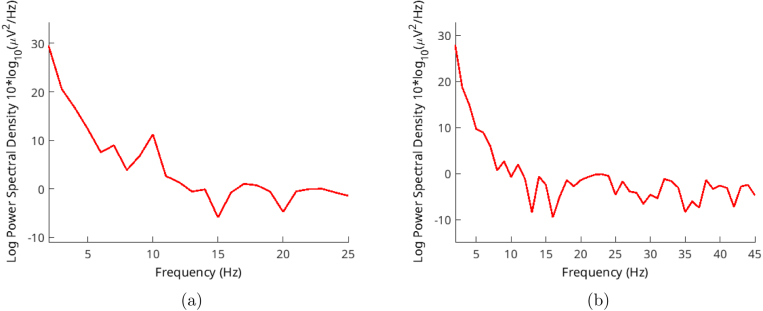
Volunteer-2 alpha test for (a) closed (b) open eyes.

Here, Fig. 10 shows alpha spectral analysis of volunteer-1. A noticeable peak exists on 10 Hz frequency. For the same records Fig. 11 shows spectrum of the signal with dominating 50 Hz AC noise. Alpha wave can spread around 8–12 Hz. Fig. 12 shows volunteer-2 results. These frequency peaks are useful for BCI systems.

**Fig. 13:**
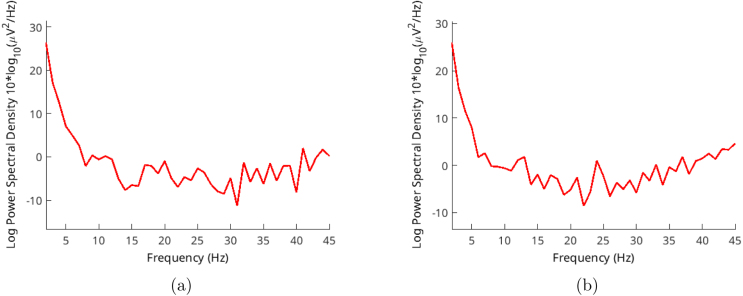
Volunteer-1 (a) 20 Hz - (b) 24 Hz SSVEP test.

**Fig. 14:**
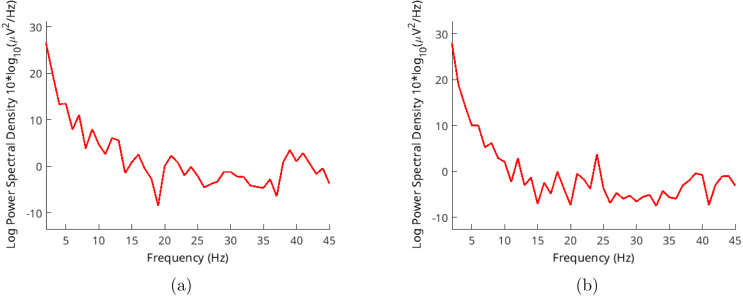
Volunteer-2 (a) 22 Hz - (b) 24 Hz SSVEP test.

**Fig. 15:**
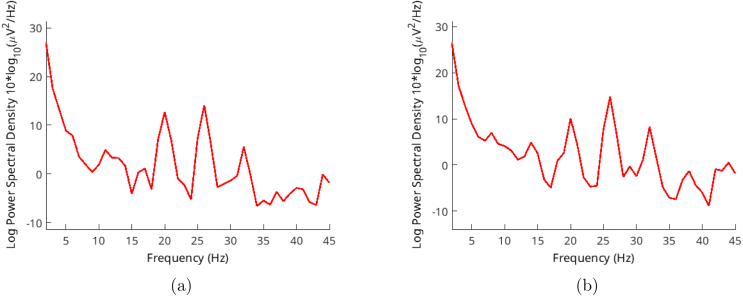
Volunteer-3 (a) 20 Hz - (b) 26 Hz SSVEP test.

Another method for validation involves SSVEP stimulations. These stimulations share a flicker frequency and can be verified by analyzing their frequency range, similar to our examination of alpha waves. We utilized random frequencies ranging from 20 to 26 Hz in steps of 2 Hz. This allowed us to ensure the functionality across different frequencies. Results are shown in Figs. 13, Fig. 14, Fig. 15.

Both signals are most pronounced in the occipital lobe of the brain, situated at the back of the head where the nerves from our eyes are connected. To capture these signals, we positioned two electrodes in the O1 and O2 spots, following the standard 10–20 system.

Volunteer-3 was exclusively subjected to SSVEP stimulations. Surprisingly, despite having long and dense hair, this individual exhibited noticeable peaks at the designated frequencies. The light stimulation shares the same ground with the device, causing both signals to appear in the outcomes depicted in Fig. 15.

## Ethics statement

This device bears a resemblance to a class-1 medical device, designed solely for educational and experimental use. The hardware’s design was centered around adhering to biomedical safety standards. The participants in this study are researchers specializing in electronic and biomedical engineering. They are fully informed about the nature of this medical device and have been briefed on the procedures involved in this experiment.

Prior to commencing the hardware verification stage, volunteers were given comprehensive information and provided with informed consent. For the scope of this study, it is essential to note that the volunteers had no known medical history related to epilepsy and exhibited no scars in the operational areas.

## CRediT authorship contribution statement

**M. Kancaoğlu:** Writing – review & editing, Writing – original draft, Visualization, Validation, Software, Resources, Methodology, Data curation. **M. Kuntalp:** Supervision, Project administration, Funding acquisition.

## Declaration of competing interest

First author of this article is a PhD candidate who has received 100/2000 scholarship, granted by the Turkish government to promote specific topics in PhD programs. All the hardware parts used in this study, was purchased by the first author with the support of this scholarship.
